# Psychometric Properties of the Persian Version of the Strength‐Based Parenting Scale in an Adolescent Sample

**DOI:** 10.1002/brb3.70213

**Published:** 2024-12-31

**Authors:** Ahmad Asgarizadeh, Omid Shokri

**Affiliations:** ^1^ Faculty of Education and Psychology Shahid Beheshti University Tehran Iran

**Keywords:** factorial validity, positive parenting, psychometric properties, strength‐based parenting

## Abstract

**Purpose:**

Despite the increasing interest in positive psychology and the functional characteristics of one of its practical derivations, strength‐based parenting, there is a paucity of information regarding the assessment tools for strength‐based parenting and their psychometric properties. Thus, this study aimed to translate the Strength‐Based Parenting Scale (SBPS) into Persian and investigate its validity and reliability among Iranian adolescents.

**Method:**

Of the 645 adolescents who completed the Persian translation of the SBPS (349 females, *M*_age_ = 14.01 years, *SD*_age_ = 0.82, range = 13–15), 300 also answered the Mental Toughness Scale for Adolescents and the Connor‐Davidson Resilience Scale.

**Finding:**

Confirmatory factor analysis supported a two‐factor structure comprising dimensions of “knowledge of strengths” and “use of strengths,” the measurement invariance of which was established across sexes and school grades. The two dimensions were significantly and strongly correlated with the constructs of mental toughness and psychological resilience (*r* = 0.36–0.65, *p* < 0.01), corroborating the criterion‐related validity of the SBPS. Furthermore, excellent internal consistency coefficients were observed for both subscales (0.96–0.97).

**Conclusion:**

Our findings substantiate the positions derived from positive psychology, upon which the conceptual foundation of strength‐based parenting is built, and provide preliminary support for the psychometric properties of the Persian version of the SBPS.

## Graphical abstract

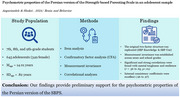

## 1 Introduction

A vast body of research has demonstrated that parents play an essential role in shaping the psychological capital of their adolescents (Arslan, Burke, and Majercakova Albertova 2022). The context of the parent–adolescent relationship and the quality of dyadic interpersonal interactions are influenced by various personal and contextual factors (Donato and Bertoni 2017; Gulliford et al. 2015). Upon embarking on adolescence and facing an accumulation of vicissitudes in various aspects of social and personal life, adolescents are challenged to adaptively manage the overload of demands resulting from diverse developmental tasks (Loton and Waters 2017; Waters 2020). Over the past half‐century, researchers have authoritatively explored the functional characteristics of parenting styles in the context of parent–adolescent relationships; however, in the latest findings stemming from the principles of positive psychology, Waters (2015a, 2015b) highlights that strength‐based parenting (SBP) is state of the art, since it promotes positive parental behaviors.

Parenting practices are traditionally defined by the two dimensions of warmth and strictness, through which four parental styles emerge: authoritative (characterized by high warmth and strictness), indulgent (characterized by high warmth and low strictness), authoritarian (characterized by low warmth and high strictness), and neglectful (characterized by low warmth and low strictness) (Baumrind 1978; Darling and Steinberg 1993; Garcia et al. 2020; Maccoby and Martin 1983; Martinez‐Escudero et al. 2020). The beneficial effects of authoritative parenting have been extensively studied (Dosman and Gallagher 2022; Morris et al. 2021; Smetana 2017); however, Waters (2015a, 2015b, 2017) recently proposed a third dimension, developmental focus, suggesting that parenting practices can also be placed on a spectrum ranging from deficit‐focused to strength‐focused. Parents with a deficit‐based developmental approach aim to mitigate their child's perceived weaknesses to prevent potential limitations in adulthood, whereas a strength‐based approach emphasizes identifying and cultivating the child's inherent strengths to foster adaptive development and future well‐being. Waters (2015a, 2015b) found empirically that the strength‐based approach explained a significant part of teenagers' life satisfaction beyond authoritative parenting and argued that it adds a “positive filter” to adolescents' stress‐coping ability.

This most recent perspective on parenting research revolves around the concept of “flourishing” (Waters 2020). Flourishing involves considering the psychological capabilities of adolescents, as well as identifying and nurturing their unique and specific strengths. In other words, fostering intellectual and psychological strengths in adolescents is a vital prerequisite for flourishing at both the micro and macro levels, which warrants the prioritization of SBP. It is argued that pursuing adolescents' psychological empowerment within the context of parenting is likely to be feasible by focusing on the elements of SBP. This will, in turn, provide the foundation for the ultimate goal, which is for adolescents to flourish (Bullen et al. 2023).

The SBP approach focuses on identifying and nurturing positive capital, states, and processes in offspring (Waters 2015a, 2015b) and includes strategies parents adopt for guidance in achieving and enriching their strengths, irrespective of the strength type (Waters et al. 2019). Emphasizing the cultivation of strengths in adolescents leads them to utilize these strengths more effectively (Jach et al. 2018; Waters 2015a). Adolescents who take advantage of these strengths also report higher levels of life satisfaction and well‐being (Waters 2015b). Findings of numerous studies have indicated that, for adolescents, SBP is associated with higher levels of perseverance, engagement, and academic performance (Amani, Nazifi, and Sorkhabi 2020; Arslan, Allen, and Waters 2022), psychological resilience, mental toughness (Larijani, Shokri, and Sharifi 2023), socio‐emotional well‐being, school belonging (Arslan, Allen, and Waters 2022; Sumargi and Giovanni 2021), self‐efficacy, post‐traumatic growth (Zavala et al. 2022), and lower levels of psychological distress (Loton and Waters 2017).

The SBP is relatively recent; nevertheless, it has been increasingly studied by positive psychologists in different cultural contexts, whose theoretical orientation underpins this approach (Loton and Waters 2017; Sağkal and Özdemir 2019; Waters 2020). According to Govindji and Linley (2007), SBP is a bidimensional construct. The first dimension can be understood as knowledge of strengths. This dimension focuses on an individual's ability to identify and recognize their strengths accurately. The second dimension, strengths use, emphasizes the behavioral aspect of strengths. It reflects the degree to which individuals integrate their strengths into different situations. This application of strengths is, in turn, linked to experiencing more positive emotions. However, despite the existing conceptualizations and the upward trend of studies focused on positive parenting behaviors, studies on the psychometric properties of the tools that measure the characteristics of this parenting style are scarce. Among the few relevant studies, Jach et al. (2018) recently developed the Strength‐Based Parenting Scale (SBPS). In their study, the construct validity of the SBPS was supported by finding a two‐factor structure, including knowledge of strengths (SBP‐Knowledge) and the use of strengths (SBP‐Use). SBP‐Knowledge, for instance, may reflect parents' effortful and inquisitive observations of children's performance in different contexts to identify their qualities. On the other hand, SBP‐Use may entail enrolling children in extracurricular activities they are interested in and gifted in.

### 1.1 Current Study

Due to the lack of measures for assessing SBP in the nonwestern, educated, industrialized, rich, and democratic (WEIRD; Henrich, Heine, and Norenzayan 2010) context of Iran, the present study aimed to translate the SBPS into Persian and evaluate its factorial structure, measurement invariance, and criterion‐related validity in a sample of adolescents. Measurement invariance is particularly tested across sexes and school grades, as previous studies have demonstrated that not only may parents employ dissimilar practices with regard to their children's sex (Endendijk et al. 2016) and age (Shamah 2011) but also that boys and girls, as well as older and younger children, perceive parenting differently (Helwig et al. 2014; Smetana, Robinson, and Rote 2015; Sorbring, Rödholm‐Funnemark, and Palmérus 2003).

The current study potentially contributes to SBP research and practice in Iran since the limited inclusion of non‐WEIRD populations in psychology research, as well as the culture‐specificity of effective parenting practices (Turner et al. 2020), emphasize the need for culturally adapted SBP measurement and practice. Moreover, it is essential to consider the context of strengths and employ a contextualized strength‐based assessment approach (Bozic, Lawthom, and Murray 2018), which further necessitates the investigation of SBP within the Iranian cultural context. Congruent with previous findings (Jach et al. 2018; Sağkal and Özdemir 2019), we hypothesized to (1) replicate the original two‐factor structure of the SBPS, (2) establish its measurement invariance across sexes and school grades, and (3) support its criterion‐related validity through associations with mental toughness and resilience.

## 2 Methods

### 2.1 Participants and Procedure

In confirmatory factor analysis, a sample size to free parameter ratio greater than ten is suggested as an acceptable rule of thumb (Kline 2023; Kyriazos 2018). Since the two‐factor structure yields 29 free parameters, a minimum sample size of 290 participants was determined a priori. A total of 645 adolescents were recruited (349 females, *M*_age_ = 14.01 years, *SD*_age_ = 0.82, range = 13–15), 211 of whom were in the seventh grade, 216 were in the eighth grade, and 218 were in the ninth grade. Sex‐specific sociodemographic characteristics are presented in Table 1. A subset of this sample (*n* = 300, 165 females; *M*_age_ = 14.03 years, *SD*_age_ = 0.83, range = 13–15; 98 seventh graders, 96 eighth graders, and 106 ninth graders) also completed measures of mental toughness and resilience (see Measures).

**TABLE 1:** Sex‐specific sociodemographic characteristics of participants.

Characteristics	Girls (*n* = 349)	Boys (*n* = 296)
	Frequency (percentage)	
7th grade	118 (33.8)	93 (31.4)
8th grade	121 (34.7)	95 (32.1)
9th grade	110 (31.5)	108 (36.5)
	Mean (standard deviation)	
Age	13.98 (0.81)	14.05 (0.82)

The research protocol received ethical approval from the designated Ethics Committee of the Educational District in Tehran and the Educational Deputy of the Faculty of Psychology at Shahid Beheshti University. Being enrolled in one of the seventh, eighth, or ninth grades in junior high school was the inclusion criterion, and not providing informed consent (by parents and/or the students) and leaving items unanswered were the exclusion criteria. To control for sample heterogeneity and sampling variation, we recruited students from a single educational district in Tehran (District 11). Several schools within this district were chosen conveniently and approached by the researchers. Before initiating the data collection, official permission was obtained from each school's administration. Parents were informed about the objectives of the study through the school staff and the online national student system and subsequently granted their consent. After explaining the purpose of the study to the students, assuring the confidentiality of responses, and obtaining informed consent, the questionnaire battery was distributed to students. Students voluntarily agreed to complete the battery of pen‐and‐paper measures and could terminate their participation at any stage. In each school class, approximately 5% of students declined to participate. Notably, none of the responses were excluded due to the presence of missing values.

### 2.2 Measures

#### 2.2.1 Strength‐Based Parenting

The SBPS (Jach et al. 2018) was developed based on previously proposed frameworks (Govindji and Linley 2007; Waters 2015a, 2015b) and reflects adolescents' perceptions of parenting practices. This scale consists of 14 items, which are responded to on a seven‐point scale ranging from 1 = *strongly disagree* to 7 = *strongly agree*. The two‐factor structure, including SBP‐Knowledge and SBP‐Use, has been suggested for the original version (Jach et al. 2018), as well as its cultural adaptations (Sağkal and Özdemir 2019). The criterion‐related validity of the SBPS has been supported through its direct associations with positive affect, subjective happiness, mental toughness, life satisfaction, extraversion, and school engagement and its inverse associations with negative affect, neuroticism, psychological distress, and school burnout (Jach et al. 2018; Sağkal 2019; Sağkal and Özdemir 2019). Furthermore, Jach et al. (2018) reported an internal consistency coefficient of 0.95 for both SBP‐Knowledge and SBP‐Use.

This study utilized the translation/back‐translation procedure to develop the Persian version of the SBPS. First, the original English version was translated into Persian. Second, to maintain linguistic and conceptual equivalence, another bilingual individual translated the Persian translation back into English. Subsequently, the two translators discussed the existing incongruities, and through an iterative review process, these differences were minimized to the extent possible. The Persian translation is available in Table S1.

After preparing the Persian translation of the SBPS, in order to examine the content and face validity, five faculty members who specialized in positive psychology studies were surveyed about the appropriateness of the items for Iranian adolescents. The opinions indicated the suitability of the Persian translation. In the next step, the researchers asked 20 adolescent boys and girls to respond to the items of the scale on a 5‐point scale from *completely comprehensible* to *completely incomprehensible*. The results demonstrated that all participants unanimously evaluated the 14 SBPS items as *comprehensible* or *completely comprehensible*.

#### 2.2.2 Mental Toughness

The Mental Toughness Scale for Adolescents (MTS‐A; S. McGeown, St. Clair‐Thompson, and Putwain 2018) is an 18‐item scale that measures mental toughness (e.g., *I am happy to try new and challenging tasks*) and is rated on a 4‐point scale ranging from 1 = *strongly disagree* to 4 = *strongly agree*. The scale is composed of six dimensions, including challenge, commitment, emotion control, life control, confidence in abilities, and interpersonal confidence. The MTS‐A was associated with motivation, nonadaptive thoughts and behaviors, generalized anxiety, test anxiety, and depression, supporting its criterion‐related validity (S. McGeown, St. Clair‐Thompson, and Putwain 2018). The internal consistency of the MTS‐A has been found to be satisfactory (Dagnall et al. 2019; S. McGeown, St. Clair‐Thompson, and Putwain 2018). The validity and reliability of the Persian version of the MTS‐A have also been supported in a sample of gifted Iranian adolescents (Khodaei 2022). In this study, Cronbach's alpha and McDonald's omega for the total score were 0.86.

#### 2.2.3 Resilience

The Connor–Davidson Resilience Scale (CD‐RISC; Connor and Davidson 2003) measures psychological resilience using 25 items (e.g., *When things look hopeless‚ I don't give up*) and five subscales: personal competence, tolerance of negative affect, positive acceptance, self‐control, and spiritual influence. Respondents rate each item on a five‐point scale ranging from *not true at all* to *true nearly all of the time*. Several studies have found evidence for the validity and reliability of the CD‐RISC (Windle, Bennett, and Noyes 2011), as well as its Persian version (Nooripour et al. 2022). In this study, the Cronbach's alpha and McDonald's omega for the total score were 0.75.

### 2.3 Data Analysis

#### 2.3.1 Data Screening

All analyses were conducted using the lavaan (v. 0.6‐16; Rosseel 2012) and semTools (v. 0.5‐6; Jorgensen et al. 2022) packages in R (R Core Team 2023), as well as IBM SPSS v. 27 (IBM Corp 2020). Descriptive statistics were examined for individual items prior to factor analysis. Absolute skewness and kurtosis values smaller than 2 and 7, respectively, indicated the absence of non‐normality (Kim 2013), and corrected item‐total correlations larger than 0.30 demonstrated satisfactory inter‐correlations between items (de Vaus 2013).

#### 2.3.2 Factor Analysis

We tested the first hypothesis by examining the original two‐factor structure of the SBPS. Since this theoretical, a priori structure was suggested by the developers, confirmatory factor analysis was employed. The robust maximum likelihood (MLR) estimator was chosen as it is appropriate for Likert‐type scales with five or more response categories (Li 2016; Rhemtulla, Brosseau‐Liard, and Savalei 2012) and robust against multivariate non‐normality (Morin 2023).

The decision on the retention of a model was made considering model fit, standardized factor loadings, and reliability coefficients. Multiple indices were used to evaluate the goodness of fit of the proposed models: the Comparative Fit Index (CFI), Tucker–Lewis Index (TLI), Root Mean Square Error of Approximation (RMSEA), and Standardized Root Mean Squared Residual (SRMR). The chi‐square (*χ* ^2^) significance level has been criticized due to its over‐sensitivity to sample size and minor misspecifications (Marsh, Hau, and Grayson 2005); hence, we did not use it and reported the values solely for transparency. Model fit was considered acceptable provided that CFI/TLI ≥ 0.90, RMSEA ≤ 0.08, and SRMR ≤ 0.10 (Pituch and Stevens 2016). Additionally, standardized loadings (*λ*) ≥ 0.30 were deemed meaningful (Morin, Myers, and Lee 2020). Correlated factors reliability coefficient, model‐based omega, and multidimensional alpha (values ≥ 0.70 deemed acceptable) were also utilized to indicate the reliability of the factors (Cho 2022; Kalkbrenner 2023).

Furthermore, we employed CFA to compare the previously suggested two‐factor measurement model against a single‐factor counterpart, reflecting SBP as a unidimensional construct. These solutions were compared using the Akaike Information Criterion (AIC), the Bayesian Information Criterion (BIC), and the sample‐size adjusted BIC (SABIC). All three indices penalize for model complexity and are used for contrasting non‐nested models, where lower scores suggest improved model fit (Kline 2023; Meyers, Gamst, and Guarino 2017).

#### 2.3.3 Measurement Invariance

To test the second hypothesis, we assessed measurement invariance separately for sex (female and male) and for school grades (seventh, eighth, and ninth grades) following a stepwise approach (Brown 2016). First, individual models were evaluated for each group (i.e., one model for each sex and one for each grade) to confirm acceptable fit within each group. Upon establishing an acceptable fit, we proceeded with testing configural invariance to assess structural equivalence across groups. Following this, additional constraints were sequentially applied to evaluate metric (weak) invariance by constraining factor loadings, scalar (strong) invariance by constraining intercepts, and strict invariance by constraining residuals. Fit indices of each step were contrasted with those of the previous step, and invariance was established provided that there was a ΔCFI/ΔTLI ≤ −0.01 complemented with a ΔRMSEA ≤ 0.015 (Chen 2007).

#### 2.3.4 Criterion‐Related Validity

Pearson correlation coefficients were computed to examine the third hypothesis: aligned with previous theoretical and empirical evidence (e.g., Larijani, Shokri, and Sharifi 2023), we expected to find significant and strong associations between SBP and the relevant constructs of mental toughness and resilience. The coefficients were interpreted as small, medium, and large, respectively, for *r* = 0.10, 0.20, and 0.30 (Funder and Ozer 2019; Gignac and Szodorai 2016).

## 3 Results

### 3.1 Descriptive Statistics

Table 2 contains descriptive statistics for the individual items of the Persian version of the SBPS. No missing data were present. Skewness, kurtosis, and corrected item‐total correlation values were well within acceptable ranges. The interitem correlation matrix is available in Table S2.

**TABLE 2:** Item‐level descriptive statistics.

#	Items	Mean	Standard deviation	Min‐max	Skewness	Kurtosis	CITC
1	My parents see the strengths (personality, abilities, talents, and skills) that I have. والدینم از توانمندی‌های من مانند استعدادها، مهارت‌ها و ویژگی‌های شخصیتی‌ام، آگاه‌اند.	4.18	1.96	1–7	−0.19	−1.25	0.80
2	My parents don't know what my strengths are. والدینم با توانمندی‌ها و قابلیت‌های من آشنا نیستند.	4.12	1.90	1–7	−0.10	−1.07	0.74
3	My parents know what I do best. والدینم می‌دانند که من چه کاری را بهتر می‌توانم انجام دهم.	4.02	1.96	1–7	−0.11	−1.24	0.86
4	My parents are aware of my strengths. والدینم از توانمندی‌هایی که من دارم، آگاه‌اند.	4.02	1.98	1–7	−0.08	−1.27	0.91
5	My parents know the things I am good at doing. والدینم می‌دانند که من در انجام چه کاری، مهارت بیشتری دارم.	3.97	2.00	1–7	−0.08	−1.33	0.90
6	My parents know my strengths well. والدینم به‌خوبی توانمندی‌هایم را می‌شناسند.	3.92	1.94	1–7	−0.02	−1.24	0.90
7	My parents see the things I do best. والدینم کارهایی را که می‌توانم به خوبی انجام دهم، می‌بینند.	3.95	2.03	1–7	−0.05	−1.33	0.88
8	My parents give me opportunities to regularly do what I do best. والدینم همیشه به من این فرصت را می‌دهند تا آنچه را که در انجامش موفقم، انجام بدهم.	3.98	2.01	1–7	0.02	−1.29	0.88
9	My parents encourage me to always play to my strengths. والدینم مرا تشویق می‌کنند، تا همیشه در انجام کارها از توانمندی‌های خود استفاده کنم.	4.15	2.11	1–7	−0.11	−1.38	0.91
10	My parents encourage me to do what I'm good at. والدینم مرا تشویق می‌کنند تا کارهایی را انجام دهم که در انجام آنها مهارت دارم.	4.06	2.12	1–7	−0.08	−1.42	0.91
11	My parents suggest I should use my strengths every day. والدینم تاکید می‌کنند که باید هر روز در انجام کارهایم از توانمندی‌های خود استفاده کنم.	4.09	1.94	1–7	−0.08	−1.24	0.81
12	My parents give me lots of opportunities to use my strengths. والدینم فرصت‌های زیادی را برایم ایجاد می‌کنند تا از توانمندی‌های خود استفاده کنند.	3.93	2.03	1–7	0.03	−1.30	0.87
13	My parents help me think of ways to use my strengths. والدینم به من کمک می‌کنند تا درباره نحوه بکارگیری توانمندی‌هایم، بیندیشم.	3.94	1.97	1–7	0.01	−1.23	0.89
14	My parents show me how to use my strengths in different situations. والدینم به من نشان می‌دهند که چگونه می‌توانم از توانمندی‌های خود در موقعیت‌های مختلف استفاده کنم.	3.97	2.01	1–7	0.01	−1.30	0.88

### 3.2 Factor Analysis

**FIGURE 1:**
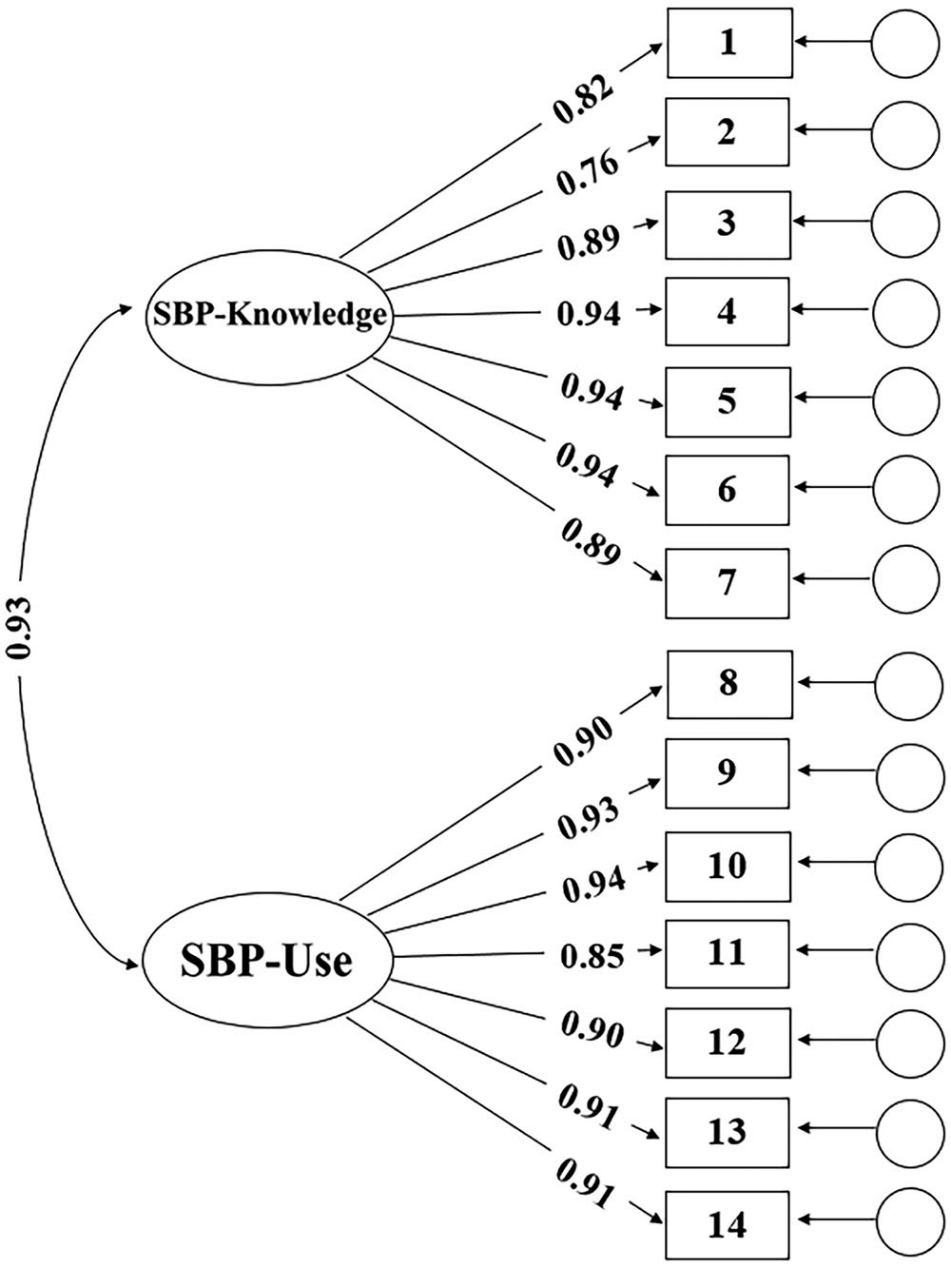
The two‐factor confirmatory structure of the SBPS.

Progressing with the confirmatory factor analysis, the single‐factor solution demonstrated a mediocre fit to the data, while the previously suggested two‐factor solution, including SBP‐Knowledge and SBP‐Use, yielded an acceptable fit; the model comparisons also favored the two‐factor solution (Table 3; ΔAIC = −534.16, ΔBIC = −529.69, ΔSABIC = −532.86). Thus, we proceeded with the evaluation of parameter estimates for the two‐factor solution (Table 4). For both subscales, standardized factor loadings were statistically significant (*p* < 0.001) and exceeded the acceptable threshold (SBP‐Knowledge: *λ* = 0.76–0.94, *M*_λ_ = 0.88; SBP‐Use: *λ* = 0.85–0.94, *M*_λ_ = 0.91). Furthermore, the reliability coefficients of the factors were identical across the three estimation methods (SBP‐Knowledge: *α* = 0.96, *ω* = 0.96, *ρ* = 0.96; SBP‐Use: *α* = 0.97, *ω* = 0.97, *ρ* = 0.97). Thus, the two‐factor solution was retained for all further analyses (Figure 1). Thus, the two‐factor solution was replicated, and the first hypothesis was supported.

**TABLE 3:** Fit indices and the range of factor loadings for the tested models.

Models	*χ* ^2^ (df)	CFI	TLI	RMSEA [90% CI]	SRMR	AIC	BIC	SABIC
Single‐factor	503.07 (77)	0.930	0.917	0.093 [0.087–0.098]	0.031	26,884.56	27,009.70	26,920.80
Two‐factor	226.35 (76)	0.975	0.970	0.055 [0.049–0.062]	0.019	26,350.40	26,480.01	26,387.94

**TABLE 4:** Standardized factor loadings of the two‐factor solution.

	SBP‐Knowledge		SBP‐Use			
Items	*λ*	SE	*λ*	SE	*δ*	*R* ^2^
1	0.817	0.049	—	—	0.333	0.667
2	0.756	0.059	—	—	0.428	0.572
3	0.891	0.046	—	—	0.205	0.795
4	0.937	0.038	—	—	0.121	0.879
5	0.935	0.039	—	—	0.125	0.875
6	0.938	0.040	—	—	0.120	0.88
7	0.891	0.044	—	—	0.206	0.794
8	—	—	0.901	0.043	0.188	0.812
9	—	—	0.934	0.040	0.128	0.872
10	—	—	0.936	0.037	0.125	0.875
11	—	—	0.846	0.048	0.285	0.715
12	—	—	0.896	0.045	0.197	0.803
13	—	—	0.914	0.041	0.165	0.835
14	—	—	0.910	0.040	0.172	0.828

Abbreviations: δ = residual variances (uniquenesses), λ = standardized factor loadings, SE = standard error.

### 3.3 Measurement Invariance

For the measurement invariance tests, we first evaluated the fit of individual models for each group: females, males, and seventh‐, eighth‐, and ninth‐grade students. Each of these five single‐group models displayed acceptable fit indices (Table 5). With an adequate fit confirmed for each group, we proceeded to test configural invariance for both sex and grade. Configural invariance was established for sex (*χ* ^2^_(152)_ = 342.47, CFI = 0.971, TLI = 0.965, RMSEA = 0.062 [90% CI: 0.055–0.069], SRMR = 0.021) and grade (χ^2^_(228)_ = 527, CFI = 0.957, TLI = 0.948, RMSEA = 0.078 [90% CI: 0.071–0.085], SRMR = 0.024), indicating that the basic structure was consistent across these groups. Subsequently, we tested metric, scalar, and strict invariance by successively adding constraints on factor loadings, intercepts, and residuals. Each model retained adequate fit without significant declines (Table 5), supporting the second hypothesis (i.e., invariance across both sex and grade at each level).

**TABLE 5:** Measurement invariance across sexes and grades.

Model	*χ* ^2^ (df)	CFI	TLI	RMSEA [90% CI]	SRMR	S–B *χ* ^2^ (Δdf)	ΔCFI	ΔTLI	ΔRMSEA	ΔSRMR
**Sex**										
Females	162.07 (76)	0.978	0.973	0.057 [0.047–0.066]	0.019	—	—	—	—	—
Males	180.57 (76)	0.962	0.954	0.068 [0.058–0.078]	0.027	—	—	—	—	—
Configural	342.47 (152)	0.971	0.965	0.062 [0.055–0.069]	0.021	—	—	—	—	—
Metric	361.47 (164)	0.970	0.967	0.061 [0.054–0.068]	0.026	11.45 (12)^ns^	−0.001	0.001	−0.001	0.005
Scalar	393.18 (176)	0.967	0.966	0.062 [0.055–0.068]	0.028	31.45 (12)^*^	−0.003	−0.001	0.001	0.002
Strict	450.44 (190)	0.960	0.962	0.065 [0.059–0.071]	0.028	47.56 (14)^*^	−0.007	−0.004	0.003	0
**Grade**										
7th	199.90 (76)	0.946	0.935	0.088 [0.076–0.100]	0.027	—	—	—	—	—
8th	196.53 (76)	0.955	0.946	0.086 [0.074–0.098]	0.026	—	—	—	—	—
9th	133.58 (76)	0.971	0.965	0.059 [0.046–0.072]	0.025	—	—	—	—	—
Configural	527.00 (228)	0.957	0.948	0.078 [0.071–0.085]	0.024	—	—	—	—	—
Metric	564.01 (252)	0.955	0.951	0.076 [0.069–0.083]	0.036	23.40 (24)^ns^	−0.002	0.003	−0.002	0.011
Scalar	597.46 (276)	0.953	0.954	0.074 [0.067–0.080]	0.037	25.31 (24)^ns^	−0.001	0.003	−0.002	0.001
Strict	615.87 (304)	0.955	0.959	0.069 [0.063–0.075]	0.036	35.00 (28)^ns^	0.001	0.006	−0.005	0

Abbreviations: ns, nonsignificant; S–B, Satorra–Bentler scaled.

* *p* > 0.05.

### 3.4 Criterion‐Related Validity

Regarding the criterion‐related validity of the SBPS, SBP‐Knowledge and SBP‐Use yielded significant and strong correlation coefficients with mental toughness and psychological resilience (Pearson's *r* ranging from 0.36 to 0.65; Table 6); hence, corroborating our third hypothesis. Specifically, greater knowledge of children's strengths and facilitating the use of these strengths were strongly associated with greater levels of mental toughness and psychological resilience.

**TABLE 6:** Descriptive statistics and Pearson correlation coefficients for the study variables (*n* = 300).

Variables	SBP‐Knowledge	SBP‐Use	Mental toughness	Psychological resilience
SBP‐Knowledge	1			
SBP‐Use	0.80	1		
Mental toughness	0.65	0.53	1	
Psychological resilience	0.40	0.36	0.62	1
Mean (SD)	35.08 (9.18)	35.05 (9.57)	48.74 (5.47)	65.05 (15.63)

*Note*. All coefficients are significant at *p* < 0.01.

## 4 Discussion

This study aimed to investigate the psychometric properties of the Persian version of the SBPS in Iranian adolescents. Our findings were consistent with those of Jach et al. (2018) and thus with the first hypothesis, indicating that the factor structure of the SBPS consists of two dimensions: SBP‐Knowledge and SBP‐Use. Additionally, the SBPS was invariant across sexes and school grades, and associations between SBP‐Knowledge, SBP‐Use, and related constructs of mental toughness and psychological resilience provided further evidence in support of its criterion‐related validity. SBP‐Knowledge and SBP‐Use also demonstrated excellent internal consistency, suggesting the SBPS to be a reliable measure.

The theoretical foundation of the SBP reflects a higher level of conceptual complexity by including a component of strength‐based processes in addition to strength‐based knowledge and use (Waters 2015a, 2015b); however, the adapted framework underlying the SBPS relies solely on the latter two (Govindji and Linley 2007). The correspondence between our findings and that of Jach et al. (2018) provides evidence supporting the theoretical underpinnings of the SBP and its two‐factor structure. Thus, knowledge about and use of these strengths are likely to be the two essential components of SBP. In line with our second hypothesis, we also found the SBPS to be invariant across sexes and school grades, suggesting it is safe to assume that any between‐group differences observed regarding these variables reflect actual differences rather than the potential measurement bias of the instrument.

Regarding the criterion‐related validity of the SBPS, SBP‐Knowledge and SBP‐Use were directly associated with mental toughness, supporting the third hypothesis. This is consistent with previous findings (Larijani, Shokri, and Sharifi 2023; Sağkal 2019; Sağkal and Özdemir 2019), indicating that SBP practices could contribute to equipping adolescents with positive psychological capital, such as mental toughness. SBP practices provide the necessary foundation for the emergence of mental toughness: (1) commitment or pursuing self‐affirmed goals despite potential obstacles, (2) rising to the challenges or the individual's willingness to perceive difficulties as opportunities for self‐growth, (3) emotional control or the ability to regulate emotional arousal, (4) perceived control over life or a future‐oriented outlook implying an individual's capacity to shape their future, (5) confidence or believing in the capabilities of oneself, and (6) interpersonal confidence or the ability to demonstrate assertiveness in social situations. Hence, adolescents whose parents practiced SBP are likely to exhibit greater focus on self‐selected goals, cope more effectively with challenges and pressures in both academic and nonacademic settings, adaptively regulate their emotions in stressful situations, have a greater sense of agency, demonstrate greater self‐assurance, establish more positive relationships with peers and significant others, and manage situational demands more effectively (Gulliford et al. 2015; Sumargi and Giovanni 2021; Zavala et al. 2022). In other words, the SBP approach emphasizes that experiences and facilitative environments that encourage recognition and utilization of strengths effectively cultivate mental toughness (Gordon and Gucciardi 2011; S. McGeown et al. 2017).

We also found direct relationships between the dimensions of the SBPS and psychological resilience, further corroborating our third hypothesis. As mentioned earlier, SBP, in particular, is argued to have vital impacts on adolescents' developmental outcomes. Researchers interested in exploring the conceptual correlates of the SBP aim to clarify its ability to predict adolescents' coping resources and positive capital. Described as a series of dichotomies, SBP is proposed to foster optimistic attributions over pessimistic ones, empowering interpretations rather than disempowering ones, adaptive emotion regulation as opposed to maladaptive emotion regulation, a growth mindset as opposed to a fixed mindset, self‐compassion rather than self‐criticism, self‐forgiveness over self‐condemnation, positive self‐talks instead of negative self‐talks, an ability to learn from adverse events rather than succumbing to learned helplessness, self‐efficacy over self‐doubt, and social development goals as opposed to social demonstration goals (Gulliford et al. 2015; Loton and Waters 2017; S. P. McGeown, St. Clair‐Thompson, and Clough 2016; O'Byrne et al. 2022; Sağkal 2019; Sağkal and Özdemir 2019; St. Clair‐Thompson et al. 2015).

In summary, SBP may provide children and adolescents with the opportunity to navigate challenging experiences adaptively by fostering a sense of self‐worth, self‐efficacy, and purposefulness; nurturing a resilient and empowering mindset; facilitating effective regulation of emotions, cognitions, and behaviors; and cultivating mental toughness (Arslan 2023; Arslan and Coşkun 2022a, 2022b; Arslan and Wong 2023; Sağkal 2019; Sağkal and Özdemir 2019). Thus, our findings and previous evidence collectively suggest that, through these mechanisms, SBP may enable adolescents to thrive by learning from challenging experiences, which are perceived as opportunities for growth.

Our findings provide preliminary support for the psychometric properties of the Persian version of the SBPS, suggesting the tenability of the conceptual positions derived from positive psychology, which has led to the development of the SBPS. Positive psychotherapists interested in promoting interpersonal skills in parent–adolescent relationships can utilize the Persian version of the SBPS to identify adolescents' perceptions of how their parents employ strength‐based practices and adjust their treatment goals and course accordingly. However, a number of limitations and subsequent research recommendations should be noted. First, the current form of SBPS asks adolescents to report on the practices of “parents” rather than inquiring about mothers and fathers separately. Reformulating the items in future studies may lead to identifying discrepancies in the parenting practices of mothers and fathers. Second, the information was obtained solely from self‐reports and from adolescents, potentially resulting in common method bias and inflation of the statistical correlations. Third, schools and participants were selected using a nonprobability convenience method and from a single district in Tehran. Thus, further research utilizing probability/random sampling methods could promote the generalizability of the findings. Fourth, this study investigated the reliability of the SBPS using internal consistency coefficients. Assessing other types of reliability (e.g., test–retest) should be prioritized in future studies. Finally, the present study solely focused on constructs theoretically related to SBP in positive parenting literature. Future studies may benefit from examining the associations between the SBPS and other parenting measures. Moreover, longitudinal or interventional designs studying the association between SBP practices and adolescents' developmental outcomes will be paramount.

## Author Contributions

**Ahmad Asgarizadeh**: data curation, formal analysis, writing–original draft, methodology. **Omid Shokri**: conceptualization, methodology, investigation, project administration, writing–original draft.

## Ethics Statement

Approval was obtained from the designated Ethics Committee of the Educational District in Tehran and the Educational Deputy of the Faculty of Psychology at Shahid Beheshti University. The procedures used in this study adhered to the tenets of the Declaration of Helsinki.

## Consent

Informed consent was received from all participants.

## Conflicts of Interest

The authors declare no conflicts of interest.

### Peer Review

The peer review history for this article is available at https://publons.com/publon/10.1002/brb3.70213

## Supporting information



Supporting Information

## Data Availability

The data supporting the findings of this study are available from the corresponding author upon reasonable request.
