# Supplementary material for: Psychometric Properties of the Persian Version of the Strength‐Based Parenting Scale in an Adolescent Sample
Source: Brain Behav. 2024 Dec 31;15(1):e70213. doi: 10.1002/brb3.70213 (PMC11688050; doi:10.1002/brb3.70213)
Supplement: Supplementary file 1 — Supporting Information [file BRB3-15-e70213-s001.docx]

**Supplementary Materials: Psychometric properties of the Persian version of the Strength-based Parenting Scale in an adolescent sample**

| **Table S1.** The Persian translation of the Strength-based parenting scale. | | | | | | | | |
| --- | --- | --- | --- | --- | --- | --- | --- | --- |
| # | گزاره‌ها | کاملاً مخالفم |  |  | نه موافقم نه مخالف |  |  | کاملاْ موافقم |
| 1 | پدرم و مادرم از توانمندی‌های من مانند استعدادها، مهارت‌ها و ویژگی‌های شخصیتی‌ام، آگاه‌اند. | 1 | 2 | 3 | 4 | 5 | ۶ | ۷ |
| 2 | پدر و مادرم با توانمندی‌ها و قابلیت‌های من آشنا نیستند. | 1 | 2 | 3 | 4 | 5 | ۶ | ۷ |
| 3 | پدر و مادرم می‌دانند که من چه کاری را بهتر می‌توانم انجام دهم. | 1 | 2 | 3 | 4 | 5 | ۶ | ۷ |
| 4 | پدر و مادرم از توانمندی‌هایی که من دارم، آگاه‌اند. | 1 | 2 | 3 | 4 | 5 | ۶ | ۷ |
| 5 | پدر و مادرم می‌دانند که من در انجام چه کاری، مهارت بیشتری دارم. | 1 | 2 | 3 | 4 | 5 | ۶ | ۷ |
| 6 | پدر و مادرم به‌خوبی توانمندی‌هایم را می‌شناسند. | 1 | 2 | 3 | 4 | 5 | ۶ | ۷ |
| 7 | پدر و مادرم کارهایی را که می‌توانم به خوبی انجام دهم، می‌بینند. | 1 | 2 | 3 | 4 | 5 | ۶ | ۷ |
| 8 | والدینم همیشه به من این فرصت را می‌دهند تا آنچه را که در انجامش موفقم، انجام بدهم. | 1 | 2 | 3 | 4 | 5 | ۶ | ۷ |
| 9 | والدینم مرا تشویق می‌کنند، تا همیشه در انجام کارها از توانمندی‌های خود استفاده کنم. | 1 | 2 | 3 | 4 | 5 | ۶ | ۷ |
| 10 | والدینم مرا تشویق می‌کنند تا کارهایی را انجام دهم که در انجام آنها مهارت دارم. | 1 | 2 | 3 | 4 | 5 | ۶ | ۷ |
| 11 | والدینم تاکید می‌کنند که باید هر روز در انجام کارهایم از توانمندی‌های خود استفاده کنم. | 1 | 2 | 3 | 4 | 5 | ۶ | ۷ |
| 12 | والدینم فرصت‌های زیادی را برایم ایجاد می‌کنند تا از توانمندی‌های خود استفاده کنند. | 1 | 2 | 3 | 4 | 5 | ۶ | ۷ |
| 13 | والدینم به من کمک می‌کنند تا درباره نحوه بکارگیری توانمندی‌هایم، بیندیشم. | 1 | 2 | 3 | 4 | 5 | ۶ | ۷ |
| 14 | والدینم به من نشان می‌دهند که چگونه می‌توانم از توانمندی‌های خود در موقعیت‌های مختلف استفاده کنم. | 1 | 2 | 3 | 4 | 5 | ۶ | ۷ |

| **Table S2.** Inter-item correlation matrix for the Strength-based parenting scale (*n* = 645). | | | | | | | | | | | | | | |
| --- | --- | --- | --- | --- | --- | --- | --- | --- | --- | --- | --- | --- | --- | --- |
| Items | #1 | #2 | #3 | #4 | #5 | #6 | #7 | #8 | #9 | #10 | #11 | #12 | #13 | #14 |
| #1 | - |  |  |  |  |  |  |  |  |  |  |  |  |  |
| #2 | .721^**^ | - |  |  |  |  |  |  |  |  |  |  |  |  |
| #3 | .738^**^ | .682^**^ | - |  |  |  |  |  |  |  |  |  |  |  |
| #4 | .773^**^ | .713^**^ | .828^**^ | - |  |  |  |  |  |  |  |  |  |  |
| #5 | .741^**^ | .688^**^ | .850^**^ | .871^**^ | - |  |  |  |  |  |  |  |  |  |
| #6 | .752^**^ | .705^**^ | .839^**^ | .882^**^ | .892^**^ | - |  |  |  |  |  |  |  |  |
| #7 | .728^**^ | .647^**^ | .783^**^ | .841^**^ | .829^**^ | .823^**^ | - |  |  |  |  |  |  |  |
| #8 | .697^**^ | .648^**^ | .749^**^ | .800^**^ | .778^**^ | .798^**^ | .807^**^ | - |  |  |  |  |  |  |
| #9 | .723^**^ | .648^**^ | .768^**^ | .834^**^ | .813^**^ | .809^**^ | .827^**^ | .856^**^ | - |  |  |  |  |  |
| #10 | .727^**^ | .666^**^ | .770^**^ | .819^**^ | .818^**^ | .811^**^ | .800^**^ | .841^**^ | .904^**^ | - |  |  |  |  |
| #11 | .606^**^ | .575^**^ | .664^**^ | .723^**^ | .728^**^ | .733^**^ | .725^**^ | .744^**^ | .797^**^ | .785^**^ | - |  |  |  |
| #12 | .669^**^ | .622^**^ | .746^**^ | .776^**^ | .764^**^ | .751^**^ | .773^**^ | .811^**^ | .821^**^ | .823^**^ | .783^**^ | - |  |  |
| #13 | .695^**^ | .644^**^ | .749^**^ | .792^**^ | .799^**^ | .816^**^ | .773^**^ | .812^**^ | .823^**^ | .843^**^ | .787^**^ | .837^**^ | - |  |
| #14 | .689^**^ | .629^**^ | .741^**^ | .803^**^ | .794^**^ | .793^**^ | .767^**^ | .808^**^ | .829^**^ | .839^**^ | .767^**^ | .836^**^ | .874^**^ | - |
